# A Review of Emerging Biomarkers Connecting Diabetes and Ischemic Stroke: Implications for Early Detection and Risk Stratification

**DOI:** 10.1155/jdr/2719491

**Published:** 2026-01-20

**Authors:** Nadia Hussain, Azza Ramadan, Amal Hussain Ibrahim Al Haddad, Zina Alfahl

**Affiliations:** ^1^ Department of Pharmaceutical Sciences, College of Pharmacy, Al Ain Campus, Al Ain University, Al Ain, UAE, aau.ac.ae; ^2^ AAU Health and Biomedical Research Center, Al Ain University, Abu Dhabi, UAE, aau.ac.ae; ^3^ Department of Pharmaceutical Sciences, College of Pharmacy, Abu Dhabi Campus, Al Ain University, Abu Dhabi, UAE, aau.ac.ae; ^4^ Chief Operations Office, Sheikh Shakhbout Medical City (SSMC), PureHealth, Abu Dhabi, UAE, ssmcabudhabi.ae; ^5^ Antimicrobial Resistance and Microbial Ecology Group, School of Medicine, University of Galway, Galway, Ireland, universityofgalway.ie; ^6^ Centre for One Health, Ryan Institute, University of Galway, Galway, Ireland, universityofgalway.ie

**Keywords:** biomarkers, diabetes mellitus, endothelial dysfunction, inflammation, ischemic stroke

## Abstract

Diabetes substantially increases the risk of ischemic stroke through complex metabolic, inflammatory, and vascular mechanisms, yet early identification of high‐risk individuals remains challenging. This narrative review synthesizes emerging circulating and genomic biomarkers that illuminate the pathways linking diabetes and ischemic stroke and evaluates their potential for early detection and precise risk stratification. Systematic searches of PubMed, Scopus, and Web of Science identified 141 relevant studies examining biomarkers, genetic and epigenetic factors, or risk prediction models in adults with diabetes. Evidence highlights several biomarker domains. Inflammatory markers such as high‐sensitivity C‐reactive protein, interleukin‐6, and tumor necrosis factor‐*α* indicate immune activation driving atherogenesis and plaque instability. Endothelial markers, including endothelin‐1, soluble vascular cell adhesion molecule‐1, and asymmetric dimethylarginine, reflect endothelial dysfunction and a prothrombotic state. Metabolic indicators, notably glycated hemoglobin, adipokines, and lipoprotein(a), capture cumulative glycemic burden, adipose signaling, and inherited atherothrombotic risk. Genetic and epigenetic measures, including polygenic risk scores, microRNAs, long noncoding RNAs, and DNA methylation, quantify inherited susceptibility and molecular imprints of the diabetic environment. Renal markers such as albuminuria and reduced eGFR reflect microvascular injury and consistently associate with stroke risk. Multimarker panels and multi‐omics integration using machine learning approaches show promise for improving predictive accuracy, though standardization, external validation, and demonstration of clinical utility are needed. Integrating these biomarkers with established clinical risk factors could transform stroke prevention in diabetes from reactive to proactive, enabling personalized, mechanism‐informed strategies for early detection and risk stratification.

## 1. Introduction

Over 537 million adults globally are affected with diabetes mellitus, a condition that leads to several complications including ischemic stroke. Approximately one‐third of ischemic stroke patients have concomitant diabetes, representing significant overlapping burdens on worldwide health concerns [1]. This proportion is increasing rapidly, driven by the global diabetes epidemic associated with population aging and lifestyle‐related factors [2]. The risk of ischemic stroke in individuals with diabetes is up to twofold higher and rises progressively with increasing disease duration [1]. This dual pathology worsens clinical outcomes considerably, including higher mortality rates, increased recurrence of strokes, prolonged hospital stays, and diminished quality of life [3, 4].

The traditional risk prediction models used in clinical practice such as the Framingham Risk Score and the CHA_2_DS_2_‐VASc score have notable limitations in the diabetes population [5]. Detecting subclinical or asymptomatic cerebrovascular risk is inadequate due to the reliance of classical clinical and demographic risk factors by these tools. The predictive accuracy for stroke risk is subpar (c‐statistic generally in the 0.75–0.80 range) and rarely accounts for the unique metabolic and inflammatory milieu of diabetes [6]. Most stroke risk scores demonstrate only fair performance in diverse populations and are rarely validated specifically in diabetics. This gap leaves many high‐risk diabetic individuals unrecognized until stroke occurs. Therefore, there is a clear need for more sensitive and precise predictive approaches to enable timely intervention in this high‐risk group [7, 8].

Biomarkers are measurable indicators of biological processes and can accurately reflect underlying pathophysiological changes, helping clinicians identify high‐risk patients even before clinical manifestations arise. Biomarkers are emerging as a promising tool to bridge gaps left by conventional scoring systems and also represent a promising gateway toward precision stroke medicine [9]. Incorporating novel biomarkers into risk stratification frameworks can give more precise, individualized risk predictions tailored specifically to diabetic populations. Early detection of ischemic stroke in high‐risk populations, such as diabetic individuals, can help improve clinical outcomes and potentially reduce long‐term disabilities [10].

Diagnostic biomarkers, such as specific blood proteins and other molecular indicators, can also help differentiate stroke (ischemic or hemorrhagic) from stroke mimics. Predictive biomarkers can help predict the likelihood of hemorrhagic transformation of an ischemic stroke [11]. This precision in early identification assists the targeted management of diabetes‐related vascular complications, which may otherwise go unrecognized until significant disease progression. Emerging biomarkers, including distinct circulatory microRNAs (miRNAs) and blood‐based proteins, have shown considerable promise in identifying individuals at risk even before clinical symptoms appear. Circulating mirRNAs like miR‐423‐3p and miR‐17‐3p are differentially expressed in diabetic stroke patients, indicating their potential as early markers for stroke risk stratification [12]. Additionally, vascular stress markers such as proplatelet basic protein (PPBP) and thrombospondin‐1 (THBS1) have been shown to be effective in exposing the underlying endothelial dysfunction. Since this is a key factor in diabetic vascular complications, these biomarkers are useful indicators for identifying high‐risk individuals within this population [13]. Subtle pathophysiological changes that precede clinical ischemic events could allow for earlier intervention.

Furthermore, multibiomarker panels that have integrated blood proteins like antithrombin III and matrix metalloproteinase‐9 (MMP‐9) have helped differentiate ischemic from hemorrhagic strokes and predict ischemic events, thereby broadening the diagnostic scope for both acute and preventative applications [11]. Comprehensive panels that combine inflammatory and vascular markers would have even greater predictive power than traditional clinical indicators currently in use. Effective stratification of asymptomatic individuals by risk level provides valuable data for individualized patient management [14].

The aim of this review is to provide a comprehensive evaluation of current and emerging biomarkers linking diabetes to ischemic stroke, with a focus on their translational potential for early detection and personalized risk stratification. Biomarkers were categorized into inflammatory, endothelial, metabolic, genetic, and epigenetic groups. For each category, we examined the mechanistic pathways connecting the biomarker to diabetes‐related stroke pathophysiology, summarize recent evidence associating the biomarker with stroke risk or outcomes in diabetic populations, and assess its potential clinical utility.

## 2. Methods

We conducted a narrative review of the literature on biomarkers and risk prediction of ischemic stroke in patients with diabetes. Systematic searches were performed by NH and AR using PubMed, Scopus, and Web of Science for articles published between January 2000 and March 2025. The search strategy used combinations of keywords and Medical Subject Headings (MeSH) terms, including diabetes mellitus, ischemic stroke, biomarkers, inflammation, endothelial dysfunction, genetics, epigenetics, polygenic risk, microRNAs, metabolomics, and prediction models, combined with Boolean operators (AND, OR) to optimize retrieval as described in the Supporting Information 1.

Additional relevant studies were identified through manual screening of reference lists from selected reviews, meta‐analysis, and key original research articles. Inclusion criteria were (1) studies published in English, (2) full text availability, (3) inclusion of adult patients with diabetes, and (4) reported on biomarkers, genetic or epigenetic factors, or risk prediction models for ischemic stroke. Studies were excluded if they Supporting Information (1) focused exclusively on hemorrhagic stroke, (2) investigated paediatric populations, or (3) involved nondiabetic cohorts. Both original research studies and systematic reviews were considered.

Titles and abstracts were initially screened for relevance, followed by full‐text assessment. Data were extracted using a standardized excel form, capturing study characteristics (design, sample size, and population), type of biomarkers or risk prediction models evaluated, and key outcomes (see Supporting Information 1:Table S2 for the extraction template and example entries). Evidence was synthesized qualitatively, focusing on emerging biomarkers, predictive performance of risk models, and gaps in the current literature.

**Figure 1:**
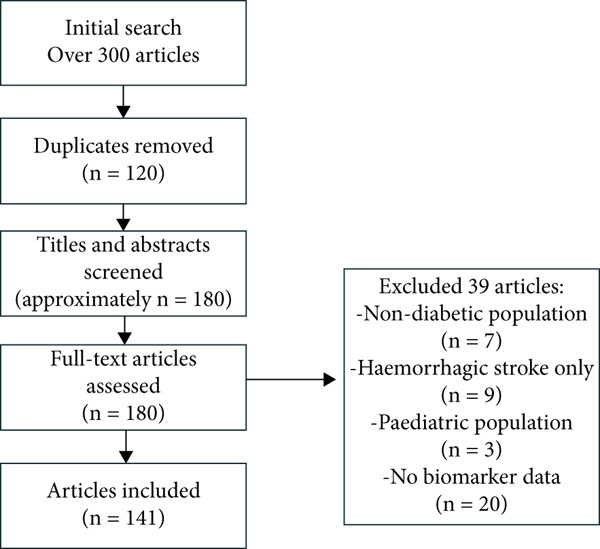
Study selection process.

The initial search yielded over 300 articles. After removing duplicates and screening titles and abstracts for relevance, approximately 180 full‐text articles were assessed. Of these, 141 peer‐reviewed studies met the inclusion criteria and were incorporated into this review. These encompassed observational cohorts, clinical trials, biomarker discovery studies, and systematic reviews. A PRISMA‐style flow diagram illustrating the study selection process is provided in Figure 1.

## 3. Pathophysiological Mechanisms Linking Diabetes and Ischemic Stroke

**Figure 2:**
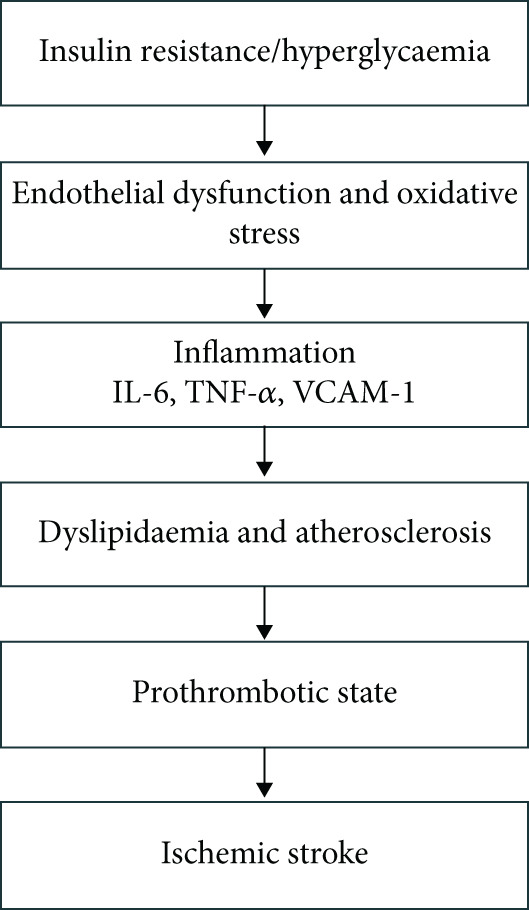
Schematic overview of how diabetes promotes ischemic stroke via endothelial dysfunction, inflammation, atherosclerosis, and thrombosis.

Diabetes increases the risk of ischemic stroke through multiple pathophysiological pathways, including chronic hyperglycemia, vascular dysfunction, and inflammation (Figure 2). Endothelial damage and disruption of the blood–brain barrier worsen stroke outcomes by amplifying inflammatory responses and is linked to recurrent episodes [15]. Concomitant atherosclerosis and hypertension, inherent to diabetes, further increase the likelihood of ischemic events. Studies have suggested glucose fluctuations and triglyceride levels as potent predictors for ischemic stroke in diabetic patients [16]. Chronic hyperglycemia accelerates atherosclerosis and contributes to vascular injury by forming advanced glycation end‐products (AGEs). These molecular products damage endothelial cells and provoke inflammatory responses, including monocyte and macrophage recruitment and cytokine release in cerebral blood vessels [17]. Microvascular structures are particularly susceptible in diabetes. Within the neurovascular unit (NVU), diabetes induces premature senescence of endothelial cells and pericytes, promoting neurovascular inflammation and compromising blood–brain barrier integrity, thereby increasing vulnerability to ischemic injury [18]. Furthermore, diabetes‐related arterial stiffness reduces vascular compliance, which can disrupt cerebral blood flow regulation, leading to white matter hyperintensities and other indicators of cerebrovascular disease [19].

### 3.1. Insulin Resistance (IR) and Vascular Dysfunction

IR plays a central role in diabetes‐related vascular dysfunction by impairing insulin′s ability to promote vasodilation and reduce inflammation. IR exacerbates endothelial dysfunction that is a recognized mechanism connected to stroke vulnerability [20]. IR impairs the phosphatidylinositol 3‐kinase (PI3K) pathway vital for nitric oxide (NO) production, and reduction in NO bioavailability promotes endothelial dysfunction. IR also upregulates pro‐inflammatory markers including interleukin‐6 (IL‐6) and high‐sensitivity C‐reactive protein (hs‐CRP), exacerbating vascular injury [21]. Additionally, IR contributes to oxidative stress and atherogenesis by increasing the production of reaction oxygen species (ROS), leading to lipid peroxidation and direct NO activation which further blunts vasodilatory capacity. Excess ROS and oxidative stress also stimulate vascular smooth muscle cell proliferation and migration, contributing to intimal thickening and atherosclerotic plaque formation [22]. Therefore, IR creates a state of chronic endothelial dysfunction, reduced cerebral perfusion, and a prothrombotic backdrop placing the brain at ischemic risk. Clinical studies have found IR is associated with greater carotid atherosclerosis and incident stroke risk [23–25].

Impairments in insulin signaling are also linked to cerebral small vessel disease and diminished vasoreactivity. This may explain the higher prevalence of lacunar infarcts and white matter lesions in diabetic individuals [1]. Additionally, biomarkers such as the triglyceride‐glucose (TyG) index, one of the measures of IR, have been independently associated with increased stroke risk, particularly in asymptomatic individuals [26]. This underscores its potential for early risk stratification and highlights that mitigating IR, a modifiable risk factor, could help reduce the burden of ischemic stroke through timely detection and intervention.

### 3.2. Chronic Inflammation, Atherosclerosis, and Immune Dysregulation

The chronic low‐grade inflammation characteristic of diabetes accelerates atherosclerosis, the key pathological process underlying most ischemic strokes. Hyperglycemia and excess free fatty acids in diabetes activate innate immune pathways, leading to increased levels of circulating cytokines (IL‐6, tumor necrosis factor‐*α*) and acute‐phase reactants. These inflammatory mediators damage the vascular endothelium and promote leukocyte adhesion and entry into the arterial intima [27]. Hyperglycemia promotes the formation of AGEs, which modify low‐density lipoproteins (LDLs) and matrix proteins, facilitate their uptake by macrophages, and activate the receptor for advanced glycation end‐products (RAGE) on endothelial cells and monocytes. This results in NF‐*κ*B signaling, cytokine release, and foam cell formation as well as smooth muscle proliferation in arterial walls, culminating in atherosclerotic plaque formation [28].

Atherosclerotic plaques in diabetes are often unstable, prone to rupture, and thromboembolism due to the increased inflammatory cell content and increased extracellular matrix [29]. Chronic systemic inflammation in diabetes also impairs plaque healing and promotes a procoagulant state (e.g., via elevated fibrinogen and plasminogen activator inhibitor‐1 [PAI‐1]), compounding the risk of plaque thrombosis [30]. Notably, a systematic review and meta‐analysis of prospective studies reported that each standard deviation (SD) increase in IL‐6 levels is associated with an approximately 19% higher risk of ischemic stroke, underscoring the role of subclinical immune dysregulation in diabetes‐related cerebrovascular disease [31]. Inflammation also damages the cerebral microcirculation, with pro‐inflammatory monocytes disrupting the blood–brain barrier and promoting small vessel disease, linking diabetes to lacunar infarcts and vascular cognitive impairment [1].

### 3.3. Endothelial Dysfunction and Coagulation Pathways

Endothelial dysfunction, driven by IR, hyperglycemia, and inflammation, is a key precursor to thrombosis and stroke in diabetes. It involves reduced production of vasoprotective mediators (NO, prostacyclin) and increased expression of adhesion molecules and tissue factor, which results in a procoagulant endothelium [32]. Persistent hyperglycemia worsens endothelial dysfunction through oxidative stress, impaired shear stress responses, and reduced endothelial progenitor cell (EPC) availability for vascular repair. It also triggers excessive endothelin‐1 (ET‐1) release, a potent vasoconstrictor that decreases cerebral blood flow and raises blood pressure, further elevating stroke risk [33].

Diabetic vasculopathy is also marked by dysregulation of the NO pathway, as is evidenced by the elevated levels of asymmetric dimethylarginine (ADMA), an endogenous inhibitor of NO synthase. This is connected to an increased risk of cerebrovascular events and worse outcomes after stroke, underscoring the role of NO pathway disruption in diabetic vasculopathy [23].

Alongside these changes, diabetes impacts coagulation and fibrinolysis. Patients with diabetes exhibit increased platelet reactivity and elevated plasminogen activator inhibitor‐1 (PAI‐1 levels, among other prothrombotic changes [34]. Clinically, patients with diabetes and atrial fibrillation have higher rates of intracardiac thrombus and acute thrombotic events. They also exhibit accelerated arterial stiffening (due to glycation of vascular collagen and smooth muscle dysfunction), which can lead to pulse pressure elevations and cerebrovascular microbleeds [35].

Overall, diabetes produces a convergence of endothelial injury and hypercoagulability that substantially heightens the likelihood of an ischemic stroke. These pathological insights provide the rationale for targeting endothelial health and inflammatory pathways, potentially through biomarkers, to identify high‐risk patients and guide preventive strategies.

### 3.4. Lipid Metabolism and Atherogenic Dyslipidemia

Lipid metabolism disturbances link diabetes to stroke, with Type 2 diabetes often causing atherogenic dyslipidemia characterized by high triglycerides, low HDL cholesterol, and small dense LDL particles, accelerating plaque formation [36]. Elevated LDL, particularly in oxidized form, is a major driver of large‐artery atherosclerosis leading to carotid and intracranial stenosis [37]. Dyslipidemia is recognized as a stroke risk factor on par with hypertension and smoking. Consistent with this, clinical trials have demonstrated that intensive lipid‐lowering therapy (e.g., with high‐dose statins or PCSK9 inhibitors) significantly reduces stroke risk in high‐LDL patients, highlighting the causal role of lipids in cerebrovascular risk [38–40].

IR in diabetes drives hepatic overproduction of very low‐density lipoprotein (VLDL) and impairs chylomicron clearance, worsening hypertriglyceridemia, endothelial injury, and vascular inflammation [41]. Elevated triglyceride‐rich lipoproteins and remnants are linked to small‐vessel strokes and white matter disease, whereas low high‐density lipoprotein (HDL) impairs reverse cholesterol transport and endothelial protection. Diabetic dyslipidemia increases both extracranial and intracranial atherosclerosis and promotes unstable plaques, raising stroke risk [42]. Lipid variability further contributes to endothelial dysfunction via inflammatory and oxidative pathways. Biomarkers reflecting dyslipidemia risk, such as apolipoproteins and lipoprotein particle profiles, may improve risk stratification [43].

In summary, diabetes promotes ischemic stroke through a multifactorial network of interrelated mechanisms: IR and hyperglycemia drive endothelial dysfunction; chronic inflammation accelerates atherosclerotic plaque formation and instability; dyslipidemia augments macro‐ and micro‐vascular damage; and prothrombotic and pro‐oxidative states heighten the likelihood of vascular occlusion. These pathophysiological links provide a basis for identifying biomarkers that capture the heightened stroke risk in diabetics.

### 3.5. Inflammatory Cytokine Expression in Lacunar Versus Non‐Lacunar Stroke Among Diabetic Patients

Recent studies highlight that inflammatory responses vary by ischemic stroke subtype in individuals with diabetes. Pacinella et al. demonstrated that pro‐inflammatory cytokines such as IL‐6, TNF‐*α*, and IL‐1*β* exhibit distinct expression profiles between lacunar (small‐vessel) and non‐lacunar (large‐vessel) strokes, suggesting different immune‐activation pathways [44]. Similarly, it is reported that diabetic patients with lacunar infarction displayed lower acute‐phase cytokine peaks but sustained elevations in endothelial‐activation markers compared with non‐lacunar cases [45, 46]. These findings imply that the inflammatory milieu in diabetes is stroke‐subtype specific, reinforcing the need for tailored biomarker panels and precision risk stratification.

## 4. Emerging Biomarkers

Emerging biomarkers are classified into categories reflecting key pathophysiological mechanisms. In this review, they are grouped as inflammatory, endothelial, metabolic, genetic, and epigenetic markers. A summary of key biomarkers, their predictive strength, and supporting evidence across populations is presented in Table 1.

**Table 1:** Summary of emerging biomarkers linking diabetes and ischemic stroke.

**Biomarker category**	**Representative markers**	**Population evidence/study type**	**Predictive ability/effect size**	**Level of evidence**
Inflammatory	hs‐CRP, IL‐6, TNF‐*α*	Multiethnic cohorts, meta‐analyses	IL‐6 associated with approximately 19% higher stroke risk per SD increase; hs‐CRP independently predicts events.	High
Endothelial	ET‐1, VCAM‐1, ADMA	European and Asian stroke cohorts	Higher ET‐1 and ADMA linked to recurrent risk; elevated VCAM‐1 associated with larger infarct volume.	Moderate–high
Metabolic	HbA1c, Lp(a), adiponectin/leptin ratio	Large T2DM prospective studies	Each 1% increase in HbA1c associated with approximately tenfold higher risk; high Lp(a) roughly doubles recurrent stroke risk.	High
Genetic/epigenetic	Polygenic risk scores, miR‐423‐3p, miR‐17‐3p	GWAS and transcriptomic studies	Polygenic risk score improves C‐statistic by 0.02–0.04; specific miRNAs upregulated in diabetic stroke	Emerging
Renal/vascular	Albuminuria, eGFR	Multicenter observational cohorts	Presence of albuminuria and reduced eGFR associated with higher cerebrovascular risk	High

Abbreviations: ADMA, asymmetric dimethylarginine; eGFR, estimated glomerular filtration rate; ET‐1, endothelin‐1; Lp(a), lipoprotein(a); PRS, polygenic risk score; SD, standard deviation; T2DM, Type 2 diabetes mellitus.

### 4.1. Inflammatory Biomarkers

#### 4.1.1. hs‐CRP

hs‐CRP is a well‐established biomarker of systemic inflammation that promotes the upregulation of adhesion molecules including vascular cell adhesion molecule‐1 (VCAM‐1) and intercellular adhesion molecule‐1 (ICAM‐1), facilitating leukocyte adhesion to the endothelium and subsequent vascular injury [21]. It also amplifies oxidative stress and impairs NO bioavailability, contributing to endothelial dysfunction and thrombogenicity [47]. Clinical studies showed that elevated levels of hs‐CRP independently predict primary and recurrent ischemic events, particularly in high‐risk populations. Each SD increase in hs‐CRP is associated with approximately a 12%–20% higher risk of ischemic stroke, independent of conventional factors [48]. Similarly, IL‐6 levels predict incident stroke, with each SD rise linked to a 19% higher stroke risk, as confirmed in meta‐analyses of multiethnic diabetic cohorts [49]. In diabetes, hs‐CRP provides incremental prognostic information when added to conventional risk factors, with consistent, albeit modest, gains in discrimination and reclassification; thus, it is a practical biomarker for incorporation into composite risk scores and monitoring strategies.

#### 4.1.2. IL‐6

IL‐6 promotes the expression of adhesion molecules (VCAM‐1, ICAM‐1) and stimulates the hepatic synthesis of acute‐phase reactants such as hs‐CRP [50]. IL‐6 has been connected to increased oxidative stress, atherosclerotic progression, and destabilization of vascular plaques. Circulating IL‐6 levels are significantly associated with ischemic stroke risk, with each SD increase in IL‐6 linked to a 19% higher incidence of stroke [31]. IL‐6 levels also have prognostic value for long‐term cardiovascular and kidney outcomes in diabetic individuals [51]. Across multiethnic cohorts and meta‐analyses, IL‐6 remains an independent predictor of incident stroke in diabetes; adding IL‐6 to clinical models yields small but reproducible improvements in risk prediction, supporting its use in multimarker panels.

#### 4.1.3. Tumor Necrosis Factor‐Alpha (TNF‐*α*)

TNF‐*α*, a central mediator of vascular inflammation and endothelial dysfunction, is usually elevated in diabetes. It upregulates VCAM‐1 and ICAM‐1, promotes leukocyte recruitment and subendothelial infiltration, and sustains inflammatory cascades that drive atherogenesis and plaque instability [52]. TNF‐*α* also increases ROS, exacerbating endothelial injury, and a prothrombotic state [53]. Elevated plasma TNF‐*α* has been associated with a greater risk of ischemic stroke in diabetic populations [17]. Although TNF‐*α* shows consistent associations with stroke risk, clinical translation currently favors measuring downstream inflammatory markers (e.g., hs‐CRP and IL‐6) for prediction; TNF‐*α* nevertheless strengthens multimarker signatures reflective of vascular inflammation.

### 4.2. Endothelial Dysfunction Markers

#### 4.2.1. ET‐1

ET‐1 is a powerful vasoconstrictor that is connected to vascular inflammation, endothelial dysfunction, and stroke pathogenesis [54]. Hyperglycemia and IR increase ET‐1 synthesis and impair its clearance [55]. ET‐1 induces ROS production, depletes NO, promotes vascular smooth muscle proliferation, and encourages leukocyte adhesion. Elevated ET‐1 levels have been linked to cerebral small vessel disease, white matter hyperintensities, and large‐artery stiffness [56]. In diabetic cohorts, higher ET‐1 and ADMA are associated with a 1.5–2‐fold higher recurrence risk of cerebrovascular events [57, 58]. In diabetic cohorts, higher ET‐1 identifies patients at elevated vascular risk and may assist in stratifying recurrent events; as a readily measurable marker, ET‐1 is suitable for inclusion within endothelial injury panels to refine risk prediction.

#### 4.2.2. VCAM‐1

VCAM‐1 mediates leukocyte adhesion to activated endothelium and plays a vital role in the initiation and progression of atherosclerosis. In diabetes, endothelial cells express higher levels of VCAM‐1 due to stimuli like hyperglycemia, TNF‐*α*, and AGE accumulation. Increased VCAM‐1 concentrations correspond to larger infarct volumes and a 30% higher likelihood of recurrent stroke [59]. Circulating soluble VCAM‐1 (sVCAM‐1) reflects the degree of endothelial activation and injury with elevated levels found in diabetic individuals, including those without overt vascular complications [60–62]. High sVCAM‐1 levels are associated with carotid intima–media thickness and correlate with other inflammatory markers such as ADMA [63].

In the context of ischemic stroke, elevated VCAM‐1 levels have been linked to increased infarct volume and worse neurological outcomes, particularly among individuals with metabolic comorbidities [59]. These findings support the role of VCAM‐1 as a biomarker of active vascular inflammation and stroke risk. Therapeutically, VCAM‐1 is being explored as a target in preclinical models, with monoclonal antibodies demonstrating atheroprotective effects by attenuating leukocyte infiltration [64]. Cross‐sectional data further suggest that diabetic individuals with macrovascular complications, including stroke, have significantly higher sVCAM‐1 levels than those without such events [62]. Prospective and stroke‐cohort data suggest that VCAM‐1 adds prognostic value beyond traditional factors; combining VCAM‐1 with inflammatory markers can improve model calibration and identify patients with active vascular inflammation.

#### 4.2.3. NO Metabolites

ADMA inhibits endothelial NO synthase and is elevated in diabetes, promoting vasoconstriction, platelet aggregation, and endothelial injury [65]. Symmetric dimethylarginine (SDMA), a structural isomer of ADMA, competes with L‐arginine uptake and similarly accumulates in diabetes. Both ADMA and SDMA are elevated following acute ischemic stroke, but their chronic elevation in diabetes likely reflects sustained endothelial stress [66].

Other NO‐related markers, such as plasma nitrite/nitrate and cyclic GMP, offer indirect measures of NO bioavailability but are less specific. Combined with markers like hs‐CRP or VCAM‐1, ADMA may improve vascular risk stratification in diabetes. ADMA levels are modifiable through lifestyle changes and certain drugs (e.g., statins and ACE inhibitors), which are linked to improved endothelial function [67].

Other endothelial markers include E‐selectin (elevated in IR), thrombomodulin (endothelial injury), and von Willebrand factor (activation from hyperglycemia). Although not stroke‐specific, they reflect systemic endothelial dysfunction. A panel of ICAM‐1, VCAM‐1, and E‐selectin has predicted Type 2 diabetes and vascular complications, suggesting a composite score could aid ischemic stroke risk prediction in diabetes [62]. Experimental markers such as circulating EPCs and endothelial microvesicles have emerged as experimental biomarkers of endothelial integrity; lower EPC counts and higher endothelial microparticles reflect endothelial stress and have been linked to worse outcomes in diabetic stroke patients [68, 69].

Although not yet ready for routine clinical use, these markers broaden the range of vascular biomarkers and offer mechanistic insight into disease progression. In summary, endothelial dysfunction markers such as ET‐1, VCAM‐1, and ADMA provide both mechanistic and clinical relevance for stroke risk prediction in diabetes. Complementing inflammatory markers, they reflect the end‐organ consequences of metabolic and immune dysregulation. Incorporating them into composite risk models could enhance precision stratification and guide individualized preventive strategies.

### 4.3. Metabolic Biomarkers

#### 4.3.1. Glycated Hemoglobin (HbA1c)

HbA1c remains a key marker of long‐term glycemic control and contributes to cardiovascular risk prediction. Chronic hyperglycemia promotes atherogenesis and prothrombotic states, linking higher HbA1c to increased stroke risk. Studies show that each 1% rise in HbA1c is linked to up to a tenfold increase in ischemic stroke risk, independent of other factors, and this association persists in diabetic patients with atrial fibrillation, even after adjusting for anticoagulation [70–72]. Individuals with prediabetic HbA1c levels (~5.7%–6.4%) also exhibit higher stroke risk compared with those with normal glycemia. HbA1c above 5.6%, the upper end of normal, predicts increased ischemic stroke likelihood, indicating that “high‐normal” glycemia may be harmful [73]. In established diabetics, there is evidence of a dose–response; in the study by Wang et al. participants in the highest quartile of HbA1c had up to a tenfold higher risk of stroke compared with those in the lowest quartile [74].

HbA1c shows paradoxical U‐shaped associations with mortality, as very low levels may reflect comorbidities or hypoglycemia risk. Nevertheless, poor glycemic control (high HbA1c) generally predicts higher stroke risk and worse outcomes, including larger infarcts and more hemorrhagic transformations [75]. Routinely measured in diabetic patients, HbA1c can be integrated into stroke risk models to identify those who may benefit from intensified glycemic control. It serves as both a risk marker and a modifiable target, with individualized goals—often < 7%—considering comorbidities and hypoglycemia risk. Although short‐term intensive glucose lowering (e.g., ACCORD) did not reduce stroke events, long‐term optimization is likely beneficial. In acute stroke, admission HbA1c correlates with severity and prognosis, with higher levels linked to worse deficits and recovery [75, 76]. Because HbA1c is routinely measured and readily interpretable, integrating it with inflammatory or endothelial markers can improve discrimination and reclassification in diabetic stroke prediction models without additional testing burden.

#### 4.3.2. Adipokines: Adiponectin and Leptin

Adipose‐derived adipokines exert systemic metabolic and vascular effects with opposing cerebrovascular roles. Adiponectin is generally anti‐inflammatory and vasoprotective, whereas leptin is pro‐inflammatory and atherogenic [77]. In Type 2 diabetes and obesity, adiponectin is paradoxically reduced despite its beneficial effects on insulin sensitivity, endothelial NO production, and cytokine suppression (e.g., TNF‐*α* inhibition) [78]. Stroke‐related findings are mixed: Some studies link higher adiponectin to worse outcomes (“adiponectin paradox”), whereas others associate low levels with atherogenesis [79]. A meta‐analysis found no overall association between adiponectin and incident stroke, though subgroup analyses suggest that in insulin‐resistant states, low adiponectin may promote stroke via dyslipidemia and inflammation. Adiponectin also inversely correlates with carotid plaque burden and thickness [80].

Leptin levels are typically elevated in obesity and Type 2 diabetes due to leptin resistance [81]. Beyond regulating energy balance, leptin stimulates the sympathetic nervous system, promotes platelet aggregation, and induces inflammatory cytokines. High leptin has been linked to endothelial dysfunction and increased carotid intima–media thickness [82]. However, in the Framingham Heart Study, baseline leptin was not associated with overall incident ischemic stroke, with an inverse relationship observed in women with high waist–hip ratios (HR per SD increase in log‐leptin ≈0.61 in the top WHR quartile) [83]. Mendelian randomization analyses (using genetic proxies for leptin) have not found a clear causal role for leptin in coronary or stroke risk (e.g., OR ≈1.48; 95% CI 0.78–2.80), suggesting leptin might be a marker of obesity rather than an independent cause [84]. A large prospective cohort found that elevated leptin was linked to higher cardiovascular risk, especially in individuals with low‐grade inflammation and poor cardiorespiratory fitness, highlighting the context‐dependent vascular effects of leptin. Diabetic dyslipidemia further increases extracranial and intracranial atherosclerosis and promotes unstable plaques, elevating stroke risk [85].

In acute stroke among patients with diabetes, lower leptin has paradoxically correlated with worse outcomes, potentially reflecting catabolic states [86]. Chronically, hyperleptinemia remains undesirable as leptin can increase tissue factor and PAI‐1 and stimulate plaque angiogenesis, promoting thrombosis and plaque vulnerability. As a biomarker, leptin may capture obesity‐related vascular risk beyond BMI, though routine testing is rare and interpretation must consider sex differences. The adiponectin–leptin ratio integrates these opposing signals, with a low ratio (low adiponectin, high leptin) indicating high cardiometabolic risk. Including adipokines in risk models could improve stroke risk prediction in diabetes, especially when obesity is a major contributor [87]. Given heterogeneity across studies, single adipokines have limited stand‐alone predictive value; the adiponectin–leptin ratio and inclusion within multimarker cardiometabolic panels may yield greater translational utility for risk prediction in diabetes.

#### 4.3.3. Lipoprotein(a) (Lp(a))

Lp(a) is an LDL‐like particle bound to apolipoprotein(a) (apo(a)) with antifibrinolytic properties. Elevated, inherited Lp(a) increases atherosclerotic risk and has emerged as a stroke marker. In stroke cohorts, Lp(a) above the 90th percentile doubled recurrent ischemic stroke risk in diabetes (OR ~ 2.06), with > 2.5‐fold higher recurrence in large‐artery stroke when combined with diabetes but showed no significant effect in nondiabetic patients [88]. These findings suggest that diabetes and high Lp(a) confer synergistic stroke risk, likely via enhanced inflammation, oxidative modification, and coexisting dyslipidemia. Measuring Lp(a) in diabetic patients could identify those needing aggressive therapy (e.g., high‐intensity statins, PCSK9 inhibitors, or Lp(a)‐lowering agents) [89]. The guidelines recommend at least one Lp(a) measurement in high‐risk or familial cases. For stroke prevention, very high Lp(a) in diabetes may warrant aspirin or targeted interventions. Observational studies, such as REGARDS, support Lp(a) as a predictor of incident ischemic stroke [90].

Lp(a) is genetically determined and stable, so a single measurement is informative. Antisense therapies targeting apo(a) in late‐phase trials can reduce Lp(a) by over 80%, highlighting its value as a precision biomarker. Elevated Lp(a) is strongly linked to higher stroke risk in diabetes, especially for recurrence, and should inform risk assessment and therapy [52]. Metabolomic profiling also offers insight into the diabetes–stroke link. Circulating branched‐chain amino acids (isoleucine, leucine, and valine), associated with IR, correlate with carotid atherosclerosis and stroke risk [91]. AGEs, measurable in plasma or via skin autofluorescence, reflect cumulative hyperglycemia and oxidative stress and are associated with arterial stiffness and stroke incidence [92]. Because Lp(a) is stable and measured once, its incorporation into diabetes risk assessments can identify high‐risk individuals for intensified lipid‐lowering or emerging Lp(a)‐targeted therapies, enhancing precision prevention.

### 4.4. Genetic and Epigenetic Biomarkers

The risk of ischemic stroke in diabetes is modulated by individual genetics as well as epigenetic modifications [93]. Tools such as genome‐wide association studies (GWAS) and epigenome‐wide analysis help unravel the cumulative effect of variants captured by polygenic risk scores (PRS) [94]. Diabetes can also imprint epigenetic changes (such as DNA methylation) that can serve as biomarkers of risk [95]. In this review, we discuss some of the most important genetic and epigenetic markers.

#### 4.4.1. Single Nucleotide Polymorphisms (SNPs) and PRS

Large scale GWAS have identified numerous stroke‐associated SNPs, including variants linked to blood pressure regulation (e.g., HDAC9), cardioembolic stroke (e.g., MITX2), as well as variants involved in inflammatory and metabolic pathways [15]. Although individually, common variants have a small effect (odds ratios ~1.1–1.3), PRS aggregating many loci helps to stratify genetic risk beyond clinical predictors. Incorporating a stroke PRS into conventional risk factors can improve prediction of incident stroke, enhancing the C‐statistic [96]. In diabetes, genetic risk interacts with the metabolic environment, producing additive and potentially synergistic effects, with the highest absolute stroke risk seen in individuals with both diabetes and high polygenic risk [97]. Additionally, certain genetic variants modify how diabetes contributes to stroke. APOE variants affect lipid metabolism and cerebral amyloid angiopathy risk, altering stroke profiles in diabetic APOE4 carriers. The 9p21 locus (CDKN2B‐AS/ANRIL) is linked to coronary and carotid disease, with its risk potentially amplified in diabetes [98]. PRS for diabetes also exist; among stroke survivors, higher genetic risk for diabetes is linked to poorer glycemic control and increased recurrent vascular events [99].

Although not yet in routine use, PRS are approaching clinical applicability and may help identify diabetic individuals with disproportionately high inherited risk, guiding the intensity of preventive strategies. Most GWAS to date are based on European‐ancestry cohorts, highlighting the need for validation in diverse and diabetes‐specific populations. PRS modestly improve discrimination over clinical models in external cohorts; current priorities are standardization, calibration across ancestries, and evaluation of clinical utility (e.g., decision‐curve analysis) before routine adoption.

#### 4.4.2. miRNAs

These small (~22 nucleotides) noncoding RNAs regulate gene expression posttranscriptionally and play key roles in cardiovascular biology and diabetes. Circulating miRNAs are stable in blood and exhibit disease‐specific signatures, supporting their potential as biomarkers. Distinct miRNA expression patterns have been observed in diabetes‐related stroke [100]. A study by Toor et al. compared circulating miRNAs in patients with acute ischemic stroke, with and without Type 2 diabetes, and found that miR‐423‐3p and miR‐17‐3p were significantly upregulated, approximately 1.8–2.3‐fold in the diabetic stroke group. Predicted target analysis linked these miRNAs to inflammation and cell death pathways [12].

Endothelial‐enriched miR‐126 associated with endothelial function and miR‐146a, an NF‐*κ*B/TLR pathway regulator, are both downregulated in diabetes, consistent with a role in reducing inflammatory signaling [101]. Other miRNAs (e.g., miR‐124 and miR‐223) correlate with stroke outcomes and are being evaluated as prognostic markers [102]. Dysregulation of miR‐146a and miR‐155 in diabetes may contribute to atherogenesis by modulating macrophage and endothelial activation [103]. Currently, measurement requires RT‐qPCR methods or sequencing with rigorous normalization and updated MIQE guidance [104]. However, the pathophysiological mechanism is compelling, since single miRNAs can report on multiple intersecting pathways. Another example is miR‐21, which is often elevated in diabetes. In carotid atherosclerosis, reviews link miR‐21 to plaque biology, and recent evidence indicates that increasing—not decreasing—miR‐21 enhances plaque stability in preclinical models [105]. Small panels of validated miRNAs may provide incremental prediction above clinical factors, but require assay standardization, external validation, and assessment of added value (C‐statistic, net reclassification) in diabetic cohorts.

#### 4.4.3. Long Noncoding RNAs (LncRNAs)

LncRNAs (> 200 nucleotides) regulate gene expression at chromatin, transcriptional, and posttranscriptional levels and are emerging players in cardiovascular disease and diabetes, with several connected to atherosclerosis and stroke [106]. ANRIL at chromosome 9p21 influences cell‐cycle regulation in the vessel wall and has strong genetic links to coronary and carotid disease as well as Type 2 diabetes [98].

Diabetic 9p21 risk allele carriers may have elevated ANRIL, accelerating plaque development. MIAT is upregulated in ischemic stroke and diabetic microvascular disease, linking to endothelial dysfunction. MALAT1, induced by diabetes, regulates vascular inflammation, microvascular integrity, and poststroke angiogenesis; higher circulating levels are associated with worse outcomes, supporting its biomarker potential [107].

Although lncRNA assays are not yet standardized for clinical use, the cellular specificity of certain lncRNAs (e.g., endothelial‐enriched transcripts) suggests diagnostic potential. H19 is downregulated in insulin‐resistant skeletal muscle and its restoration improves insulin signaling; conversely, in vascular cells H19 upregulation has been linked to neointima formation and atherosclerosis, indicating tissue‐dependent effects rather than atherogenesis driven by H19 deficiency [108, 109]. At present, lncRNAs are investigational biomarkers; their translational role will depend on analytical validity and demonstration of independent, reproducible predictive value in diverse populations.

#### 4.4.4. DNA Methylation and Other Epigenetic Marks

DNA methylation captures both inherited risk and cumulative environmental exposures, including hyperglycemia, providing a basis for “metabolic memory.” In diabetes, epigenome‐wide analyses have identified methylation patterns linked to cardiovascular risk, with changes at loci involved in endothelial function and inflammation correlating with prior cardiovascular events [15]. Epigenetic age acceleration, where methylation‐based biological age exceeds chronological age, is associated with a higher risk of diabetes complications, including stroke; each additional year of acceleration increased the odds of incident complications by 11% in one analysis. Such epigenetic clocks (e.g., the 7‐CpG clock) could thus help predict which diabetic individuals are prone to complications like stroke [110].

Specific gene methylation is also informative. Hypomethylation of blood pressure–related genes such as HDAC9, PDE3A, and PRDM6 is associated with higher ischemic stroke risk, and adding these methylation markers to traditional factors improved prediction; individuals with multiple hypomethylated loci also had higher 3‐month post‐stroke mortality [111]. Global hypomethylation (e.g., in repetitive elements) appears more common among diabetics who develop cardiovascular events, which suggests genomic instability. Epigenetic “risk scores” that integrate methylation across multiple CpGs are under development; a methylation score predicted coronary heart disease in one cohort and offers a model for stroke risk in diabetes [112]. Although cost and technical complexity currently limit routine implementation, blood‐based methylation assays are feasible, and markers such as LINE‐1 methylation (a proxy for global methylation) have been linked to stroke incidence [113].

Genetic and epigenetic biomarkers offer complementary perspectives on diabetes‐related stroke risk. SNPs and PRS capture inherited susceptibility and interindividual variation, whereas epigenetic markers such as circulating mirRNAs, lncRNAs, and DNA methylation reflect dynamic gene regulation influenced by the diabetic milieu. Integrating these genomic and molecular signals with clinical and protein biomarkers enables a more comprehensive, mechanism‐informed approach to risk stratification and personalized prevention. Emerging epigenetic risk scores that combine methylation data across loci show promise but require rigorous evaluation using transparent reporting frameworks and clinical utility analyses before adoption in routine prediction.

## 5. Clinical Implications and Risk Stratification

### 5.1. Integration of Biomarkers Into Risk Prediction Models

Integrating molecular biomarkers with established clinical risk factors can enhance individual risk assessment and guide targeted preventive strategies. Contemporary stroke risk calculators (e.g., the Framingham Stroke Risk Profile, QRISK, and ASCVD estimators) perform only modestly in external validations and rarely incorporate diabetes‐relevant biomarkers (pooled C‐statistic ~0.78), limiting discrimination in heterogeneous diabetic populations [7]. Augmenting such tools with orthogonal biological signals is a route that can be considered. Adding an inflammatory marker (e.g., hs‐CRP or interleukin‐6) together with an endothelial or vascular marker (e.g., soluble VCAM‐1 or ADMA) could identify diabetic individuals whose conventional factors would only place them at intermediate risk. This approach is already in use in cardiovascular medicine; the Reynolds Risk Score improved coronary risk prediction by incorporating hs‐CRP with standard factors [114]. In atrial fibrillation, the biomarker‐based ABC‐stroke score, combining age, NT‐proBNP, high‐sensitivity cardiac troponin, and clinical history of prior stroke or transient ischemic attack, demonstrated better discrimination than CHA_2_DS_2_‐VASc in both the derivation cohort (C‐index 0.68 vs. 0.62) and the external validation cohort (0.66 vs. 0.58) [115].

In diabetes care, kidney injury measures are already recognized as vascular risk markers: albuminuria and estimated glomerular filtration rate (eGFR) add much needed prognostic information for cardiovascular events beyond traditional factors [116, 117]. These examples demonstrate that a few carefully selected and validated biomarkers can markedly enhance risk prediction, improving both calibration and discrimination.

Effect sizes for inflammatory biomarkers vary by population and endpoint. GlycA and hs‐CRP show distinct associations with cardiovascular outcomes: GlycA predicts myocardial infarction more strongly, whereas hs‐CRP relates more to ischemic stroke, highlighting outcome‐specific rather than uniform predictive performance [118]. Quantitatively, the inclusion of GlycA or hs‐CRP alongside conventional risk factors improves model discrimination modestly, with C‐statistic increases of approximately 0.01–0.03 and net reclassification improvements of 2%–4% [119, 120]. In diabetic populations, hs‐CRP retains independent predictive value for stroke after adjustment for established vascular risk factors, suggesting additive benefit when incorporated into multimarker prediction models [121]. Earlier community data from the Northern Manhattan Study reported that hs‐CRP did not independently predict incident stroke after multivariable adjustment, highlighting cohort‐level differences [122]. These data emphasize that biomarker‐based refinement of risk scores offers measurable, albeit incremental, and gains in predictive power.

Economic evaluations of biomarker‐guided prevention remain limited but increasingly supportive. Analyses incorporating inflammatory and renal biomarkers into cardiovascular risk prediction models suggest that selective testing may be cost‐effective when used to refine treatment allocation in high‐risk populations. For example, the addition of hs‐CRP to standard risk assessment improved event prediction at an incremental cost‐effectiveness ratio, primarily through more efficient statin allocation [123, 124]. Similarly, albuminuria screening for vascular risk stratification in diabetes has been shown to be cost‐effective due to its low assay cost and strong prognostic value [125]. These findings suggest that selective, evidence‐based biomarker testing can enhance risk prediction and optimize resource use, particularly when integrated into existing diabetes care pathways.

In general populations, adding hs‐CRP or fibrinogen to traditional risk models provides only modest benefit, with meta‐analyses showing ~ +0.004 in C‐index and ~1.5% net reclassification improvement. Consistent with the ASCVD guidelines, certain biomarkers serve as risk enhancers to refine treatment decisions, including hs − CRP ≥ 2 mg/L and Lp(a) ≥ 50 mg/dL (or ≥125 nmol/L), thresholds supported by recent guidance and cohort studies [126, 127].

### 5.2. Biomarker Panels for Early Detection

Given the multifactorial mechanisms linking diabetes to ischemic stroke, single biomarkers are likely insufficient. Multimarker panels combining inflammatory, endothelial, and metabolic signals appear more effective. Proof‐of‐concept studies in poststroke prognosis show that composite biomarker indices can enhance risk stratification beyond clinical variables, supporting this panel approach even if the specific markers differ for incident‐risk prediction [14].

For primary prevention in diabetes, a practical panel could include HbA1c (glycemic control), an inflammatory marker (hs‐CRP or IL‐6), an endothelial marker (sVCAM‐1 or ADMA), kidney function (albuminuria and/or eGFR), and a lipoprotein measure (Lp(a)). These markers are measurable on automated platforms, with weights derived from prospective cohorts using penalized regression to reduce overfitting [128, 129]. Any composite must demonstrate consistent discrimination and calibration across derivation and validation cohorts, improve net reclassification beyond clinical scores, and show clinical utility by guiding interventions that improve outcomes [70].

### 5.3. Personalized Risk Assessment and Preventive Strategies

Biomarker‐integrated models enable stratified prevention. In Type 2 diabetes, albuminuria (uACR) and reduced eGFR independently mark elevated cardiovascular risk and should trigger aggressive, multifactorial management beyond traditional factors. Contemporary guidelines explicitly link higher albuminuria/lower eGFR categories with greater cardiovascular risk and use these measures to guide treatment intensity [130, 131].

Among statin‐treated patients, hs‐CRP identifies residual inflammatory risk more strongly than LDL‐C for future events. Moreover, anti‐inflammatory therapy with low‐dose colchicine reduces major adverse cardiovascular events (including stroke) in appropriate secondary‐prevention populations (e.g., chronic/acute coronary disease), supporting targeted use when inflammation remains high [121]. In AF, adding NT‐proBNP and high‐sensitivity troponin to age and clinical history in the ABC‐stroke scheme improves discrimination and clinical decision support versus CHA_2_DS_2_‐VASc, with recent external validations in emergency department and screening contexts [132].

More broadly, policies that already acknowledge biomarker “risk enhancers” in primary prevention (e.g., hs‐CRP and Lp(a)) offer a template for integrating diabetes‐relevant stroke biomarkers into shared decision‐making. Primary‐prevention guidance already recognizes selected biomarkers—hs − CRP ≥ 2 mg/L and Lp(a)—as risk‐enhancing factors to refine treatment thresholds. This provides a template for integrating diabetes‐relevant stroke biomarkers into shared decision‐making [126].

In summary, integrating emerging biomarkers with established clinical risk factors has the potential to markedly improve early detection and risk stratification of ischemic stroke in diabetes, supporting a precision medicine approach by targeting interventions to the patients most likely to benefit. Prior to routine clinical implementation, further validation in diverse populations and interventional studies is required to demonstrate that biomarker‐guided strategies translate into improved outcomes. Nonetheless, the evidence trajectory indicates that biomarker integration is set to transform stroke risk assessment and prevention in the expanding global population of individuals with diabetes.

## 6. Current Challenges

Variability between studies, overlap among inflammatory markers (e.g., IL‐6 and hs‐CRP), and differences in assay methods for emerging targets (such as circulating miRNAs) complicate the translation of associations into practical prediction tools. Contemporary population data indicate that IL‐6 may outperform hs‐CRP for cardiovascular risk, yet the two are correlated, highlighting concerns about redundant information [133]. For mirRNAs, recent reviews highlight methodological heterogeneity (platforms, preanalytics, and timing) that hinders reproducibility and threshold setting [134]. Many biomarker studies are cross‐sectional or have not been externally validated, and decision‐curve analysis to gauge clinical net benefit is still rarely used. Current reporting guidelines clearly call for both external evaluation and assessment of real‐world clinical value [135, 136].

Accordingly, large cohorts/biobanks with adjudicated cerebrovascular outcomes and stored biospecimens are important to benchmark candidates, harmonize assays, and set cut‐offs that are clinically meaningful (e.g., UK Biobank outcome adjudication work; guidance on when formal adjudication is needed in stroke trials) [137, 138]. Cost‐effectiveness and equity, including access to advanced assays and the responsible use of genetic information, should accompany technical validation and implementation [139]. Importantly, negative or context‐dependent findings must shape use: hs‐CRP does not uniformly improve stroke prediction across populations, as shown in older community cohorts and in more recent analyses in which incremental discrimination gains were small and even inconsistent [138, 140].

Despite these challenges, progress is being made. Biomarker‐guided trials are ongoing, for example, studies testing anti‐inflammatory therapies in patients with elevated CRP (such as CANTOS and COLCOT in coronary disease), approaches that could be adapted for stroke. As evidence accumulates, the guidelines may increasingly incorporate biomarkers; the American Heart Association has already noted in a scientific statement that risk assessment in diabetes may include biomarkers such as coronary calcium or CRP in selected patients [1].

Overall, the evidence supports a staged approach: develop and validate concise, diabetes‐specific multimarker panels grounded in mechanistic biology; demonstrate incremental predictive value over existing risk models; and evaluate whether biomarker‐guided interventions improve patient‐centred outcomes. With rigorous validation and careful clinical implementation, biomarkers have the potential to advance stroke prevention in diabetes toward truly personalized risk stratification.

## 7. Recommendation for Future Directions

### 7.1. Advances in Biomarker Discovery and Validation

Advances in technology and multicenter collaboration are accelerating biomarker discovery and translation. Nonetheless, validation in large, diverse, and prospective cohorts is essential to ensure findings generalize across different ancestries and healthcare systems. Many existing stroke risk models demonstrate only fair discrimination and limited external validation, highlighting the need for rigorously tested, biomarker‐enhanced approaches [7].

Cohorts with stored biospecimens and long‐term follow‐up enable unbiased assays, such as proteomic or metabolomic screens, linked to adjudicated endpoints. Equally important are studies connecting biomarkers to specific stroke mechanisms, as diabetes influences both large‐artery atherosclerosis and small‐vessel disease. For clinical application, prospective preregistered analyses with defined thresholds, calibration assessment, and net reclassification reporting will be essential.

### 7.2. Integration of Omics Technologies (Genomics, Proteomics, and Metabolomics)

Modern omics technologies now allow simultaneous measurement of thousands of molecular features, enabling network‐level exploration of diabetes–stroke biology. High‐throughput proteomics have identified circulating proteins associated with future cardiovascular events, including growth/differentiation factor‐15 (GDF‐15) and other inflammatory mediators [141]. Emerging large‐scale proteomic studies in stroke suggest that composite protein signatures may improve prognostic stratification and potentially predict future risk. Complementary metabolomic analyses highlight pathways such as branched‐chain amino acid metabolism and the gut microbiota–derived metabolite trimethylamine‐N‐oxide (TMAO) as contributors to vascular risk [142]. Collectively, these findings support the utility of multiomic panels in capturing diverse aspects of disease biology. Critical next steps include validating candidate markers with independent methods, pre‐specifying analyzable panels, and demonstrating that omics‐based scores enhance predictive power beyond established clinical factors.

### 7.3. Artificial Intelligence (AI) and Machine Learning (ML) in Biomarker Analysis

ML methods can capture nonlinear relationships and interactions among large sets of biomarkers and clinical variables. In middle‐aged and older adults, adding inflammatory cytokines to conventional risk factors within ML frameworks has improved stroke risk stratification compared with traditional models, highlighting the potential for data‐driven selection of streamlined biomarker panels [143]. Imaging‐augmented models offer additional predictive potential. In the UK Biobank, deep‐learning–derived retinal “age gaps” from fundus photographs predicted incident stroke, indicating that vascular imaging phenotypes can complement blood‐based biomarkers [144].

As ML and AI tools approach clinical implementation, transparent reporting—following the TRIPOD‐AI guidelines—and rigorous validation are essential to minimize bias and prevent overfitting. Independent assessment of risk of bias and applicability using tools such as PROBAST should become standard practice [145].

### 7.4. Toward Precision Prevention

Biomarker‐enhanced scores could help tailor preventive therapy in diabetes by identifying people with active inflammatory, endothelial, or metabolic injury who might otherwise appear similar on traditional risk assessment. Evidence that targeting inflammation can lower vascular event rates comes from large coronary disease trials—canakinumab in CANTOS and low‐dose colchicine in COLCOT and LoDoCo2—although their impact on stroke specifically has been modest or neutral, and applying these findings to primary stroke prevention will require dedicated studies [146].

Glucagon‐like peptide‐1 receptor agonists (GLP‐1 RAs) lower the risk of incident stroke by around 16% in cardiovascular outcome trials and may be especially advantageous for patients with elevated inflammatory or atherogenic biomarkers [147]. In contrast, sodium–glucose cotransporter‐2 (SGLT2) inhibitors show no significant effect on overall stroke risk but consistently provide cardiovascular and renal benefits, including reduced heart failure and kidney events [148].

Kidney biomarkers also deserve attention in diabetes: Albuminuria and reduced eGFR—markers of endothelial and microvascular injury—are independently linked to stroke and may enhance risk prediction when included in models [149].

Lp(a), a stable, genetically determined biomarker, is another strong candidate for diabetes‐specific stroke risk panels. Potent Lp(a)‐lowering drugs—antisense oligonucleotides and small interfering RNAs—can reduce levels by 80%–95% in Phase 2 trials, and large Phase 3 studies (Lp(a)HORIZON for pelacarsen; OCEAN(a)‐Outcomes for olpasiran) are underway to determine whether lowering Lp(a) translates into fewer major events, including ischemic stroke [52].

### 7.5. Ethical, Equity, and Implementation Considerations

Equitable deployment of biomarker‐based prediction tools requires that discovery and validation cohorts represent global diversity. Without such representation, models—particularly those incorporating polygenic risk—risk exacerbating existing health disparities [150]. For emerging biomarkers, including mirRNAs and LncRNAs, standardized assays, strict quality control, and clinically meaningful thresholds are essential. Validation should also include cost‐effectiveness analyses to determine whether biomarker‐guided care offers advantages over standard practice.

Clear clinical pathways must define which patients to test, how frequently to reassess results, and how findings will guide management, whether through therapy intensification, targeted imaging, or modified follow‐up intervals. Randomized trials comparing biomarker‐driven strategies with usual care are needed to demonstrate improved outcomes and establish the evidence base for guideline integration.

Overall, advances in multiomics, ML, and rigorous validation frameworks are bringing biomarker‐guided precision prevention for diabetes‐related stroke closer to clinical reality. Key next steps include standardizing measurements, validating models across diverse populations, and conducting interventional studies to confirm that biomarker‐informed care can reduce stroke risk beyond current standards.

## 8. Conclusions

Diabetes and ischemic stroke are linked through overlapping metabolic, inflammatory, and vascular pathways. Emerging biomarkers provide insight into these mechanisms and enable earlier, more precise risk stratification. Inflammatory markers (e.g., CRP, IL‐6, and TNF‐*α*) capture immune‐driven atherogenesis and plaque instability, whereas endothelial markers (e.g., ET‐1 and adhesion molecules) reflect vascular injury, thrombogenic potential, and microvascular dysfunction. Metabolic biomarkers, including HbA1c, adipokines, and Lp(a), convey cumulative glycemic, adipose, and lipoprotein‐related risk. Genetic and epigenetic measures—such as PRS, circulating noncoding RNAs, and DNA methylation—add an individualized layer by quantifying inherited susceptibility and the lasting molecular impact of diabetes.

Integrating these markers into multibiomarker panels or ML models improves risk discrimination beyond conventional clinical variables and may identify high‐risk individuals before stroke onset. Specific markers, such as pentraxin‐3 and composite inflammatory indices, also have prognostic value for functional outcomes and early recurrence. Widely available assays—hs‐CRP, Lp(a), albuminuria, and eGFR—can already guide preventive management and enhance risk prediction in diabetes.

Translation into routine practice will require prospective validation across diverse populations, assay standardization, and demonstration of clinical benefit. Nonetheless, the evidence supports a precision medicine approach: Combining biomarkers with established risk factors and modern analytics has the potential to shift stroke prevention in diabetes from reactive to proactive, targeting interventions to those at highest biological risk while avoiding overtreatment in lower‐risk individuals.

## Conflicts of Interest

The authors declare no conflicts of interest.

## Author Contributions

**Nadia Hussain:** conceptualization, methodology, investigation, data curation. **Azza Ramadan:** visualization, writing – original draft, writing – review and editing. **Amal Hussain Ibrahim Al Haddad:** validation; writing – review and editing. **Zina Alfahl:** conceptualization, project administration, writing – review and editing.

## Funding

No funding was received for this manuscript.

## Supporting information


**Supporting Information** Additional supporting information can be found online in the Supporting Information section. PRISMA checklist and all related supporting information are provided in the supporting material.

## Data Availability

Data sharing not applicable to this article as no datasets were generated or analyzed during the current study.
