# Supplementary material for: A Review of Emerging Biomarkers Connecting Diabetes and Ischemic Stroke: Implications for Early Detection and Risk Stratification
Source: J Diabetes Res. 2026 Jan 20;2026:2719491. doi: 10.1155/jdr/2719491 (PMC12817486; doi:10.1155/jdr/2719491)
Supplement: Supplementary file 1 — Supporting Information Additional supporting information can be found online in the Supporting Information section. PRISMA checklist and all related supporting information are provided in the supporting material. [file JDR-2026-2719491-s001.zip › Hussein et al. supplementary material (tracked changes).docx]

**A Review of Emerging Biomarkers Connecting Diabetes and Ischemic Stroke: Implications for Early Detection and Risk Stratification**

**(Supplementary material)**

Nadia Hussain,^1,2^ Azza Ramadan, ^2,3^ Amal Hussain Ibrahim Al Haddad,^4^ Zina Alfahl,^5,6*^

^1^ Department of Pharmaceutical Sciences, College of Pharmacy, Al Ain campus, Al Ain University, 64141, Al Ain, United Arab Emirates

^2^ AAU Health and Biomedical Research center, Al Ain University, Abu Dhabi, UAE

^3^Department of Pharmaceutical Sciences, College of Pharmacy, Abu Dhabi campus, Al Ain University, 112612, Abu Dhabi, United Arab Emirates.

^4^ Chief Operations Office, Sheikh Shakhbout Medical City (SSMC), PureHealth, Abu Dhabi, UAE

^5^ Antimicrobial resistance & Microbial ecology group, School of Medicine, University of Galway, Galway, Ireland

^6^ Centre for One Health, Ryan Institute, University of Galway, Galway, Ireland

*Corresponding author at:

Antimicrobial Resistance and Microbial Ecology Group, School of Medicine, University of Galway, Galway, Ireland. Email address: [zina.alfahl@universityofgalway.ie](mailto:zina.alfahl@universityofgalway.ie)

**S1: Search Keywords**

Systematic searches were performed across PubMed, Scopus and Web of Science. Search strategies combined Medical Subject Headings (MeSH) and free-text terms using Boolean operators (AND, OR) to maximise retrieval.

Search terms used:

*Core terms*:* “diabetes mellitus” OR “type 2 diabetes” OR “hyperglycaemia”

*Stroke-related terms**:* “ischaemic stroke” OR “cerebral infarction” OR “lacunar stroke” OR “non-lacunar stroke”

*Biomarker-related terms:* “biomarker” OR “inflammatory marker” OR “endothelial dysfunction” OR “genetic” OR “epigenetic” OR “microRNA” OR “polygenic risk” OR “metabolomic” OR “prediction model”

*Search filters:* English language, human studies, adult populations (≥18 years).
Additional articles were identified through manual screening of reference lists from included reviews and key primary studies.

* Search terms focused on type 2 diabetes because it represents the predominant form of diabetes in adults and accounts for most of the biomarker research related to ischemic stroke. While type 1 diabetes was not used as a core search keyword due to its distinct pathophysiology and limited biomarker literature in adult stroke populations, studies involving adults with type 1 diabetes would still have been captured under the broader term ‘diabetes mellitus’.** Stroke-related search terms were restricted to ischaemic stroke and its major subtypes (cerebral infarction, lacunar and non-lacunar stroke) because the review specifically targeted ischemia-related mechanisms and biomarkers. These terms align with standard clinical and MeSH classifications and ensured retrieval of the most relevant literature without broadening the search to heterogeneous or non-ischemic stroke categories.

# **S2: Data Extraction Template and Example Entries**

| **Author, Year** | **Country** | **Study Design** | **Sample Size (n)** | **Population** | **Biomarker Category** | **Specific Biomarkers / Models Evaluated** | **Key Outcomes / Associations** | **Main Findings / Summary** |
| --- | --- | --- | --- | --- | --- | --- | --- | --- |
| Pacinella *et al.,* 2025 | Italy | Cross-sectional | 240 | Diabetic and non-diabetic patients with ischaemic stroke | Inflammatory | IL-6, TNF-α, IL-1β | Cytokine levels in lacunar vs non-lacunar subtypes | Distinct cytokine expression patterns between stroke subtypes; IL-6 significantly higher in non-lacunar stroke. |
| Licata *et al.,* 2006 | Italy | Observational | 160 | Acute lacunar and non-lacunar ischaemic stroke patients | Inflammatory / Endothelial | CRP, VCAM-1, ICAM-1 | Association with diabetic state and time of onset | Stronger endothelial activation in diabetic stroke; delayed cytokine peak in lacunar infarcts. |
| Binjawhar *et al.,* 2023 | Saudi Arabia | Experimental | 120 | T2DM adults | Endothelial | Endothelin-1 (ET-1) | Relationship between ET-1 expression and oxidative stress | Hyperglycaemia upregulates ET-1 gene via epigenetic mechanisms. |
| Chen *et al.,* 2025 | China | Prospective cohort | 5,420 | T2DM patients | Metabolic | Lipoprotein(a) [Lp(a)] | Recurrent ischaemic stroke | High Lp(a) (>90th percentile) doubled stroke recurrence risk in diabetics. |
| Toor *et al.,* 2022 | Qatar | Case–control | 90 | Acute ischaemic stroke with vs without T2DM | Genetic / Epigenetic | miR-423-3p, miR-17-3p | Circulating microRNA expression | Both miRNAs upregulated in diabetic stroke; predictive AUC = 0.81. |
| Wang *et al.,* 2022 | China | Cohort | 8,650 | Adults with prediabetes and diabetes | Metabolic | HbA1c | Incident ischaemic stroke risk | Each 1% increase in HbA1c associated with ~10× higher risk. |
| Neumann *et al.,* 2021 | UK | GWAS cohort | 20,000+ | Older adults | Genetic | Polygenic risk score (PRS) for ischaemic stroke | Model discrimination (C-statistic) | Adding PRS improved C-statistic by 0.03 vs clinical model alone. |
| Tao *et al.,* 2024 | China | Cohort | 3,200 | T2DM | Renal / Vascular | Albuminuria, eGFR | Incident cerebrovascular disease | Elevated urinary ACR and reduced eGFR independently predicted stroke. |

Abbreviations: IL: interleukin; TNF-α: tumour necrosis factor alpha; VCAM-1: vascular cell adhesion molecule-1; ICAM-1: intercellular adhesion molecule-1; ET-1: endothelin-1; HbA1c: glycated haemoglobin; PRS: polygenic risk score; ACR: albumin-to-creatinine ratio; eGFR: estimated glomerular filtration rate.

Table Notes: The full data-extraction form included all 141 studies meeting inclusion criteria. Columns captured study design, sample size, biomarker type, measurement methods and outcomes.
